# Poverty as a Political Choice: a Comparative Analysis of Reports of the UN Rapporteur’s Visits to the UK and Spain

**DOI:** 10.1007/s41134-021-00179-9

**Published:** 2021-07-16

**Authors:** Ian Cummins, Emilio José Gómez-Ciriano

**Affiliations:** 1 School of Health and Society, University of Salford, Salford, England; 2 Faculty of Social Work, Castilla-La Mancha University, Cuenca, Spain

**Keywords:** Human rights, Austerity, Poverty, Citizenship

## Abstract

This paper presents a comparative analysis of two reports by the UN Rapporteur on Extreme Poverty and Human Rights, one for Spain and one for the UK. In both countries, austerity policies were introduced following the banking crisis of 2008. The UN Rapporteur reports highlight the damage that was done by welfare retrenchment. In particular, the reports document the impact of austerity on the most vulnerable individuals and communities. The paper uses Somers' (2008) conceptual model of citizenship as the basis for a comparative analysis of two reports. Somers' (2008) model of citizenship is a triadic one which sees the state, market and civil society as competing elements. Each one can serve to regulate and limit the influence or excesses of the other two. Somers argues that neoliberalism has seen the dominance of the market at the expense of the role of the state and the institutions of civil society. Austerity policies saw the market dominating. Having examined the context of the two reports and their conclusions, the paper discussed the implications for individual social workers’ practice and the role of social work as a profession in tackling poverty and marginalisation.

## Introduction

The United Kingdom (UK) and Spain are signatories of the major international human rights treaties including the *Universal Declaration of Human Rights* and the two international covenants: *The Covenant on Civil and Political Rights (CPR)* and *The Covenant on Economic, Social and Cultural Rights (ESCR).* These place duties on governments to promote, respect and meet their human rights obligations. The United Nations (UN) Special Rapporteur on Extreme Poverty and Human Rights carries out visits to countries and investigates the impact of government economic, social and welfare policies. The Rapporteur seeks to influence policy-making and raise awareness of poverty as an issue of human rights. When visiting a country, the Rapporteur governments agree to allow the Rapporteur to meet with citizens, civil society representatives, and members of the Government.

The Special Rapporteur on Extreme Poverty and Human Rights, Philip Alston, visited the United Kingdom in November 2018 and Spain in January–February 2020. The visits discussed here were the first that the Special Rapporteur had made to either country. The visits followed critical concluding observations on the failure of both countries to protect economic, social and cultural rights after both countries had been examined by the ESCR committee the UK in 2016 and Spain in 2018. To contextualise the Special Rapporteur’s reports, the paper begins with a discussion of the impact of austerity on citizenship using Somers’s (2008) model. The paper goes on to argue that living in poverty is a deeply stigmatising experience that should be viewed as issue of human rights. The impact of austerity in the UK and Spain as well as the main themes of the reports. It then uses Somers’s (2008) model to analyze how what she terms *market fundamentalism* has had a corrosive impact on citizenship. The paper concludes with a consideration of these developments for social work and social work practice arguing that austerity created increasingly challenging ethical and practice dilemmas for individual social workers and the wider profession.

## Austerity Policies in the UK and Spain

In this section, the impact of austerity policies in the UK and Spain will be outlined. In both countries, governments responded to a fiscal crisis by reducing public spending. This period of welfare retrenchment forms the backdrop to the visits undertaken by the Rapporteur.

### Austerity in the UK

Austerity policies were followed by the Coalition government in the UK from 2010. In 2008, the initial response to the banking crisis was for the New Labour government to spend huge sums of public money to bail out financial institutions. The banks were seen as “too big to fail.” In the UK, the New Labour administration also followed standard Keynesian economics by attempting to stimulate demand in the economy. These measures included a reduction in value added tax (VAT) and increased government capital spending. Coalition governments are very rare in modern UK political history. Following the 2010 General Election, a coalition of Conservatives and Liberal Democrats took office. The Coalition presented itself as a government formed in response to a national emergency. Brown (2015) noted that calls to individual sacrifice are an integral part of the discourse of the fiscal crisis as national emergency. Beatty & Fothergill (2016) claculated that welfare spending would be reduced by $27 million annuallu by 2020-2021 and would result in a recasting of the UK welfare state and the impact of these austerity cuts are racialised and genered according to an analysis by Emejulu and Bassel (2015). Crossley (2016) concluded that the largest cuts were experienced in those areas that had officially been identified as being poorer and having greatest needs.

In the UK, austerity policies were combined with a series of reforms to the UK’s notoriously complex benefit system. The most significant reform was the introduction of Universal Credit (UC). UC was introduced in 2013 and combined a range of working-age benefits into a single payment. UC is paid monthly in arrears. This means that a claimant waits one calendar month from the date they submitted an application before, assuming they are successful, the first UC payment is made. There is then a delay in the payment to reaching the claimant’s bank account. It can take up to five weeks before the first payment is received. This means that the majority of claimants are in arrears or facing financial hardship from the start of the claim. This is not accidental. The system has been marked by logistical difficulties. UC claims must be made online. The UC system operates in real time so changes to wages, very common for those in precarious employment lead to changes in UC levels. The libraries that remain open have seen a huge upsurge in customers needing support with dealing with UC online. For example, in Newcastle, staff provided assistance to nearly two thousand customers in a year around these issues.

Austerity is the culmination of trends towards a more punitive approach to welfare. This punitive approach is a key feature of neoliberalism. New rules meant that those claiming benefits were subject to sanctions imposed on claimants who do not meet conditions such as attending job centre meetings. Sanctions can include reductions in the level of benefits or in some cases the cessation of payments. The Work Capability Assessment (WCA) scheme meant that individuals who were claiming the Employment and Support Allowance (ESA) were subject to fitness to work assessments. Barr et al. (2016) concluded that WCA process was linked to 590 suicides, 279,000 additional cases of self-reported mental health problems and 725,000 additional prescriptions for anti-depressants. The WCA regime also applies to people with physical health problems. Ryan (2019) outlined the disastrous impact of austerity and welfare conditionality on people living with disabilities.

Austerity was presented by its supporters as a technocrat exercise. This cloaks the fundamental retooling of the welfare state that it entailed (Goodman, 2018).

This period of retrenchment was the most sustained cutting of social welfare provision in modern UK political history (Taylor-Gooby, 2012). It went beyond that the Thatcher Governments of the 1980s had thought politically possible (Young, 2013). The allegedly generous nature of UK welfare provision was, in this analysis, the cause of the UK’s fiscal difficulties in 2010—not the bailing out of the banks (Cummins, 2018). In fashioning what the Prime Minister termed a “smarter state” (Cameron, 2015), austerity involved the attempted recasting of the relationship between individuals, communities and the state.

### Austerity in Spain

In the period 2011–2018, successive Conservative governments in Spain followed austerity policies. Prior to the COVID-19 pandemic, 12 million people, 25% of the population were at risk of social exclusion. In Spain, 2.5 million people were living in severe poverty. The Spanish welfare model is a hybrid system similar to Mediterranean welfare models (Guillen & León, 2011; Moreno, 2009). It combines a Conservative-Bismarkian model in work and pensions, a Scandinavian model in healthcare and a liberal model in social care and social services. There is a significant shadow economy, estimated to account for 22% of GNP in 2019. Social and family values are rooted in the Catholic tradition. Prior to the 2008 banking crisis, the Spanish welfare state was placed under extreme pressure during the economic crisis of 1992–1994 (Cabrero, 1994). From July 1992 to December 1993, unemployment increased by 800.000 and GNP fell by 1%. The government devaluated the currency and implemented austerity policies. Youth unemployment rose. The impact was partially cushioned by households whose main breadwinners still had stable jobs. This period saw the increased bifurcation of the labour market with precarity becoming more widespread. The period between the end of the 1992–1994 economic crisis and the financial crisis in 2008 saw welfare retrenchment (Cabrero, 2014). However, the Socialist Party government (2004–2008) introduced key progressive social legislation. This included the 2006 Dependency Act, which included support for people living with disabilities and their family and carers.

In Spain in 2008, the collapse of the building sector and its subsidiary industries had a huge impact. Along with this, there were increasing difficulties in the access to credit and a steep rise in the rate of unemployment. As in the UK, the Spanish government initially followed a series of Keynesian style measures. These included a public investment fund to create jobs by investing in local infrastructure, and a subsidy of 400 euros per month to support the return to the labour market of long-term unemployed people (Gómez-Ciriano, 2012). There was pressure from big banks and corporations to make employment legislation more “flexible.” Reforms reduced workers’ rights making redundancies easier and cheaper. In 2010, Prime Minister Zapatero announced a range of measures to reduce public expenditure: pensions were frozen, maternity allowance reduced and the salary of public sector workers cut by 5%. A report issued by FOESSA Foundation in 2011 argued that these measures put social cohesion at risk (Laparra & Perez Eransus, 2010). In August 2011, the two main political parties, in order to avoid a bailout and pushed by the EU troika, agreed on a constitutional reform that subjected any public expenditure to a principle of budgetary stability.

In the General Election of December 2011, the Popular Party won an absolute majority. The new Government introduced a series of reforms that were replicated across the regions of Spain by the autonomous governments. As in the UK, these reforms had an impact across all areas apart from pensions. Austerity measures included reduced employment rights for workers, a reduction in unemployment benefits and the gradual increase in the retirement age from 65 to 67 years old. Increased conditionality was introduced to the welfare system. As in the UK, these measures were, in fact, building on those introduced by previous governments. Social welfare services faced significant budget cuts thousands of professionals were made redundant and some of them felt forced to emigrate. A reform of the mortgage law fueled evictions. Statistics from The General Council of Judicial Power and the Platform of support for the evicted people reveal that from 2008 to 2014, an overall of 688,280 evictions had taken place (PAH, 2020).

The overall impact of the policies and events outlined above was to undermine the foundations of the Spanish welfare state. The final observations to the fifth Spanish periodic report issued by the Committee of Economic, and Cultural Rights Committee of the United Nations in 2012, expressed concern that the most marginalised no longer enjoyed effective protection for the rights enshrined in the Covenant. The sixth periodic report issued in May 2018, when supposedly the country had overcome the crisis, acknowledged the profound impact that the international financial crisis has had on the economy and on the effective enjoyment of economic, social and cultural rights.

Alongside economic turmoil, Spain had experienced a political and constitutional crisis. In June 2018 a motion of censorship was passed against Premier Mariano Rajoy. A left, progressive government supported by Podemos, and Catalan and Basque nationalist parties took office. However, this was not a stable coalition. Three national elections took place in less than 18 months. On January 7th 2020, Pedro Sánchez was appointed as Prime Minister. This crisis meant from June 2018 to January 2020 only urgent social measures could be implemented. The parliamentary majority was not sufficient to approve a new budget. Therefore, the budget from Rajoy’s last term was followed. By the time of the Special Rapporteur’s visit there was a new government committed to reforms, which would tackle to overcome the effects of austerity policies.

## UN Rapporteur’s visits to the UK and Spain

This section will outline the main findings from the Special Rapporteur’s visit to the UK and Spain. During these visits, the Special Rapporteur met with a range of community groups, activists, academics and government officials across both countries.

### UK

In November 2018, Professor Philip Alston, United Nations Special Rapporteur on extreme poverty and human rights visited the UK. The report (Alston, 2019a) demonstrates the way that austerity policies have shredded the social welfare safety net. The Alston report argues that austerity has seen the ripping up of the post-war Beveridge social contract. Todd (2015) notes that a previous period of austerity in the UK, which followed World War II, saw the establishment of key features of the modern welfare state including the National Health Service (NHS). This modern period of austerity saw the fragmentation and marketisation of key welfare institutions such as the NHS. The report focuses on the economic impact of austerity. However, it is framed in a discourse of cultural and social rights emphasising the value of community organizations. Living in poverty is seen as a breach of human rights. On page 1, the report notes that at the time of the report, 14 million people, one fifth of the population were living in poverty. The report goes on to state that.

“For almost one in every two children to be poor in twenty-first century Britain is not just a disgrace but a social calamity and economic disaster, all rolled into one”

The Rapporteur notes the closure of 500 children’s centres in the period 2010–2015 and the closure of 340 libraries with the loss of 8,000 jobs in the period 2010–2016. These are the sorts of services that have a vital but often hidden roles in local communities. The Rapporteur highlighted that changes in legal aid have had the overall impact of effectively denying poorer people representation in key areas of public law such as family, housing and immigration (Bowcott & Duncan, 2018). Such services are of even greater importance to poorer families and communities, who are much more likely to require such assistance and support.

Alston (2019a, b) argues that austerity had led to a form of social engineering and the shredding of the post-war Beveridge social contract. Alston (2019a, b) may have a somewhat nostalgic view of post-war British community values and the generosity of the welfare state. However, he is clear that austerity policies have produced a residual welfare state that is “punitive, mean- spirited and often callous” Alston (2019a, b p3). Alston (2019a, b) highlights that UC is the first service that is “digital by default”—i.e. the whole system is online. This wrongly assumes that all claimants are digitally literate and have access to the internet. In this huge experiment of producing a digital welfare state it the most vulnerable who have been put at most risk. The use of AI is an area of concern. Eubanks (2018) demonstrates that the use of automated decision-making in social welfare can be placed is the latest in a long history of measures that profile, police and punish poor people. Alston (2019a, b) notes that the UC system identifies claimants as being in low/medium/high categories of risk—for fraud—based on algorithms. This means that their application is subject to differing levels of scrutiny and investigation. This takes place without the applicant’s knowledge and is based on a range of factors, for example, address. This is inherently discriminatory.

“digitization of welfare systems has been accompanied by deep reductions in the overall welfare budget, a narrowing of the beneficiary pool, the elimination of some services, the introduction of demanding and intrusive forms of conditionality, the pursuit of behavioural modification goals, the imposition of stronger sanctions regimes and a complete reversal of the traditional notion that the State should be accountable to the individual”

Alston (2019a, b) concludes that the rights to contest an adverse decision or seek a meaningful remedy are rendered meaningless. The costs of austerity have fallen disproportionately on the poor, women, people from racial and ethnic minorities, and people with disabilities. Alston (2019a, b) argues that these groups are further marginalised by the overall impact of austerity. These economic policies undercut and reverse the progress that had been made in creating a framework for the protection of the social, cultural and legal rights of marginalised groups. Employment has been presented as the most effective route out of poverty. However, Alston (2019a, b) highlights that because of low wages, insecure jobs and zero hour contracts, record low unemployment occurred at a time when 14 million people are living in poverty. People using food banks are, actually, often in work. The other biggest group of food bank users is those who have been subject to welfare sanctions. The reductions in social care services increase the burden on primary caregivers who are overwhelming women. UC is based on a single payment which, can entrench gendered dynamics within relationships and make women more vulnerable. These trends have been further exposed by the COVID-19 lockdown.

## Spain

The Rapporteur’s report notes that there has been a recovery since the recession and the debt crisis of the previous decade. However, one of the key themes of the report is that the benefits of this recovery have not been enjoyed across Spanish society. The report notes that the newly elected Spanish Government faces huge challenges if it is to meet the aims of its impressive social reform agenda. These include high unemployment, including chronic youth unemployment and a housing crisis, which the report describes of being “of stunning proportions.” The report contrasts the image of Spain, both at home and abroad, as a community and family oriented society with the modern reality. Poverty is deep and widespread, and the country lacks an adequate social protection system. These social divisions are reinforced by a segregated and increasingly anachronistic education system. The Rapporteur notes that Government fiscal policies provide far more benefits to the wealthy than the poor. There is an entrenched bureaucratic mentality in many parts of the government that focuses on formalistic procedures over the well-being of people. There is a no meaningful commitment to uphold people’s social rights to housing, education, and an adequate standard of living. Across a range of social indicators, Spain is ranked near to the bottom of the EU. Neoliberalism and austerity policies have fractured shared values and social solidarity. The Rapporteur reports that time and again he met people who told him that they felt that they had been abandoned.

### Poverty in Contemporary Spain

In this report, as for the one in the UK, the Rapporteur emphasised that poverty is a political choice and a clear result of the following specific fiscal policies. Spain has amongst the highest poverty rates in Europe. About 26.1% of people in Spain, and 29.5% of children, were at risk of poverty or social exclusion in 2018. The report notes the unemployment rate of 13.78 percent is more than double the rate in the EU as a whole. For young people, the situation is even worse with around 30% of under 25s being unemployed. There are substantial rates of in-work poverty, with many people working in low-paid, part-time or temporary jobs. Inequality is also shockingly high, with indicators well above EU averages. The housing crisis has been one of the most significant drivers of inequality and poverty. The Rapporteur describes visiting areas many Spaniards would not recognise as a part of their country. This includes a shantytown, which the report describes as having worse conditions than a refugee camp. There was no running water, electricity, or sanitation. Migrant workers have lived there for years without there being any improvement in these living conditions. One of the features of modern urban poverty is that it has become increasingly geographically concentrated. As well as this spatial concentration it is racialised (Wacquant, 2009a, b). The Rapporteur’s report described “closed off neighbourhoods” of concentrated poverty. These neighbourhoods lack access to basic healthcare, welfare services and even legal electricity and paved roads.

Spain faces a youth unemployment crisis. The report highlights the links between this and wider issues of inequality. In Spain, 33.7% of those with a primary education or lower were at risk of poverty or social exclusion in 2018. This compares with 12.6% of those with higher education. Investment in education as a percentage of GDP fell significantly between 2009 and 2017. Spain provides free education. However, this does not cover basic costs that are a vital part of attending school—transportation, food, books and supplies. These have risen significantly, and 32% of families face difficulty paying education costs (Save the Children, 2019). These pressures inevitably impact on the ability of children to remain in school and achieve educational qualifications. The lack of qualifications makes entry into the labour market even more difficult. Spain leads the EU in the number of children leaving school before completing their education. In 2018, 17.9% of school children did not complete their education.

The report sums the current situation up thus

The single word that I heard the most over the past two weeks is “abandoned.” People felt abandoned in a rural town without any public transportation to visit the doctor, no money to pay for private transport, and unsure if an ambulance would come when needed. Abandoned in a stigmatized low-income suburb that the police avoid. Abandoned to unscrupulous landlords, unconscionable rent raises, or unmaintained public housing. And abandoned to an arbitrary bureaucratic system that suddenly denies or revokes vital support without explanation. (Alston, 2020, p. 4)

The Rapporteur is highly critical of policymakers arguing that social and economic rights are rarely taken seriously. They may be invoked in the abstract but this does not extend to concrete action. The report bluntly states that low cost social housing is almost nonexistent and the social assistance system is broken. In fact, wealthy families benefit more from cash transfers than poor families. As in the UK, the system is increasingly punitive and difficult, if not impossible, to navigate.

The Rapporteur highlights the work that is being done by the third sector to combat the impact of unemployment and inequality. This is another feature that is common to both the UK and Spanish reports. Whilst acknowledging the vital work of deeply dedicated staff and volunteers, the report emphasises that the Spanish government has obligations to fulfil human rights. These cannot be outsourced to third sector organizations that are often underfunded and struggling to cope with increased demand for services. The valuable work that such organizations do, must be additional to concerted Government actions and policies. The crisis of poverty in Spain is such that it can only be tackled by comprehensive, systematic interventions that require the resources and organization of the state.

## Somers, Neoliberalism and Citizenship

The Rapporteur’s visits followed periods in austerity in both countries. Austerity policies saw a reduction in public services, reduced legal rights for workers and increased conditionality within the welfare system. The result in both countries was increased inequality and poverty. These policies can be viewed as key as extensions of policies that had been key features of neoliberalism. Somers (2008) argued that the neoliberal project changed the relationship between the individual and the state. The Rapporteur’s reports are now examined in the broader context of the impact of neoliberalism on citizenship and social cohesion.

Neoliberal economic and welfare policies seek to reduce the role of the state and introduce market mechanisms into the public sector. Brown (2010, p132) argues that one result is that large scale problems—unemployment, environmental problems are “*sent down the pipeline to small and weak units unable to cope with them technically, politically or financially*.” This has become even more apparent during austerity where the pressures on marginalised communities and groups have increased but resources to support individuals and families have reduced. Somers (2008) argues that neoliberal economic policies lead to outcomes that harm the economic and social interests of the majority. These include increases in inequality, economic instability, and environmental damage. In addition, reduction in public services results in the shredding of the welfare safety net that was a cornerstone of the post-World War II social democratic consensus (Giroux, 2017).

Somers’s (2008) model of citizenship is a response to the neoliberal focus on individualism. She identifies three competing elements: *state, market and civil society*. These elements regulate or limit the influence or excesses of the other two. Somers (2008) argues that neoliberalism can be understood as a form of “market fundamentalism.” She uses fundamentalism as she views the commitment to the market as having similar characteristic to a dogmatic religious belief. Neoliberalism views key institutions of the state and civil society as barriers to market mechanisms. In Somers’s (2008) triadic model, the institutions of civil society such as trade unions, social movements, and community groups have a fundamental role. They provide a form of social protection for both vulnerable individuals and communities. They are also a counterbalance to the potential excesses of both the state and the market. In the neoliberal model, these institutions are seen as interfering in the functioning of the free market. This is why they have faced such sustained political and cultural attack since the late 1970s.

Citizenship is not simply a matter of formal legal rights. It has to be viewed as a combination of rights, institutions, and social relationships that recognises that citizens are members of social and political communities (Somers, 2008). Market fundamentalism leads to a gap between a formal declaration of rights and translating them into meaningful substantive rights in practice. Somers (2008) views the way that the state abandoned poor African Americans in the wake of Hurricane Katrina in the United States as a prime example of these processes. She argues that the victims of Hurricane Katrina became internally stateless persons. Somers’s (2008) model requires a balance between the state, market, and civil society for individuals to be socially included citizens. Wright (2015) argues that this spatial representation simplifies the way that in modern capitalist societies, the market, state and civil society, are connected and enmeshed.

Somers’s (2008) concept of citizenship is rooted in the US experience where there is a residual welfare state and a wider cultural suspicion of the role of government. In countries with a more social democratically orientated approach then social welfare structures can play a role in guaranteeing social rights. The moves towards liberal democracy in the previously totalitarian societies, have seen states become members of the EU or the Council of Europe. Membership of these bodies involves a commitment to the values of the international framework of human rights. This commitment is a necessary condition for membership of the international community. It is also viewed as a mark of the progress since the end of the previous regime. Spain joined the EU in 1986. In post-Franco Spain, there has been an increasing recognition that the institutions of the state have a role to play in the respect and protection of the rights. However, there is generally a stronger recognition and protection for civil and political rights than for the protection of economic, social and cultural ones. The focus in the period of transition from dictatorship to democracy was on civil and political rights. However, as democracy became established, the focus widened to include the recognition of economic, social and cultural rights.

## Poverty, Stigma and the Discourse of Human Rights

Goffman (1963, p3) described stigma as an attribute that is “deeply discrediting.” One of the impacts of stigma is to reduce the holder or the stigmatised person “from a whole and usual person to a tainted or discounted one.” It has to be acknowledged that, for example, the “underclass” discourse has become a deeply entrenched one in media portrayals of welfare systems. Poverty should be viewed as a human rights issue. The impact of living in poverty affects all aspects of people’s lives including their physical and mental health. Alongside this, it limits the exercise of social and political rights. In Spain and the UK, austerity policies involved significant reductions in social welfare provisions. Alongside this, welfare systems became more punitive. These shifts were part of long-standing neoliberal undermining of the welfare state. Neoliberal anti-statism sees the welfare state as both overgenerous and dependency creating. It is thus, not a solution to the problems of poverty but one of the factors in its creation. Since the early 1990s, the Right has mounted a protracted “war of position” against the key features of a universalist welfare state (Garrett, 2007). Mead (1992) and Murray (1990) present poverty as a moral issue in the sense that poverty is the result of the moral failings of those living in poverty. For Murray (1990), poor people make poor choices and are then rewarded for them by the welfare state. The condition of the public finances in 2010 thus proved to be an opportunity that was too good to miss for those who had been opposed to the fundamentals of a universalist welfare system.

Clarke and Newman (2012) term the way that the fundamentally structural issues of poverty and economic inequality are transformed into a discourse of welfare dependency and the burden on the state as the “alchemy of austerity”. Mills (2018) in her analysis of the reporting of suicides linked to austerity and benefit reforms demonstrates the way that these cases are presented as individual tragedies. This approach depoliticises them. The broader context of the government policy that lie behind these cases is ignored or down played. Grover (2019) sees austerity as a manifestation of what Engels termed social murder. Social murder encapsulates the way that the lives of working class were shortened by the consequences of economic and social inequalities that are the inevitable consequence of social relations of capitalism. Cooper and Whyte (2017) describe austerity as a form of “institutional violence” carried out in a bureaucratic form. This is not to minimise the damage that it does, rather it emphasises that it occurs on a daily basis out of sight. Cooper and Whyte (2017) emphasise the brutal, violent nature of the impact of austerity but also that this represents what Nixon (2011) termed “slow violence”.

A claim for equal treatment must also carry with it a claim for redistribution of resources Fraser (1995, 2010). Redistribution means all citizens can enjoy social, cultural, and legal rights. Modern social movements have put forward claims for equality based on some aspect of identity. “Living in poverty” is not an identity that is claimed in the same way as other modern social and political identities (Fraser, 1995). Poverty is an issue of morality and human rights. It does not feature in the same way in equality claims based on identity (Sayer, 2005a, 2005b). The process of silencing and marginalising groups or individuals excludes the voices of the oppressed. The challenge to the oppression of women and racial and sexual minorities has involved not only attacks on stereotypical constructions of identity but also the creation of positive new ones. These have, of course, been led by members of those groups. These processes are much more problematic in the area of poverty.

Calls for equality are based on the language of the international human rights framework. This has the recognition of the fundamental dignity of human beings at its core. There have been challenges to the utility of the concept of dignity. It is a powerful but also a somewhat vague concept (Dworkin, 1995). Dworkin also added that any notion of human rights had to accept that dignity would be at its core. It became “a value which is held universally and applies to all human beings” (Misztal, 2013, p. 102). Sandel (2009) argues that justice requires that all human beings are afforded rights because they are human beings and thus capable of Kantian reason. It also a key idea in major world religions. For example, Catholic social teaching holds we are created in God’s image and afforded dignity on this basis. The modern use of dignity has developed from a notion that was associated with rank and status to a universalist approach. Dignity is afforded because of one’s status as a human being (Waldron, 2007). Kateb (2011) argues that in this approach dignity is based on the notion that every individual is equal, and that no species is equal to human beings.

Pinker (2008) suggests that dignity “is a squishy, subjective notion, hardly up to the heavyweight moral demands assigned to it” (p. 1). Bioethicist Macklin (2003) argued that the term was being used to block rather than further research. She suggested that “Dignity is a useless concept” (p. 1419). In the ethical field, it can be replaced by autonomy. However, a notion of autonomy surely stems from recognition of an individual’s basic humanity—a key aspect of the idea of dignity. The second challenge to the notion of the human rights discourse is most forthrightly expressed in the work of the radical French philosopher, Alain Badiou. His critique of capitalism sees it not as a progressive force that led to the establishment of liberal rights, but as a form of nihilism (Badiou, 2015). The discourse of liberal democratic citizenship and human rights masks the reality of the exploitative nature of capitalist systems. This modern discourse, based as it is on individualism, is actually an adjunct to neoliberalism. Badiou (2015) sees this discourse of rights as a form of neocolonialism. Previously, subaltern populations are only allowed to claim rights in the forms as constructed by liberal Western democracy. Lauren (2003) outlines the controversies that surrounded the Universal Declaration of Human Rights. Du Bois argued before the US Senate Foreign Committee in 1945 that the Declaration: “reflected the national interests, the economic rivalries and the selfish demands of the governments represented at San Francisco” (Lauren, 2003). Despite these controversies, Lauren (2003) acknowledges that this was the first time that international human rights had been so openly debated and discussed.

## Discussion

Somers’s (2008) model argues that full citizenship requires economic, social and cultural justice. Economic inequality denies marginalised groups the “rights to have rights.” Arendt (1948) was concerned that the Universal Declaration of Human Rights would become “a set of pleasant normative assertions.” By this she meant that these rights were meaningless unless they were guaranteed by governmental and legal frameworks. In the broader political context, Arendt (1948) argued that if an individual ceases to be seen as a citizen of a state they lose not only their civil rights in that particular state, but also their universal and inalienable human rights. For Arendt (1948) human rights are based on membership of of a political community. Therefore, Arendt suggests that there should be a human right to belong to a political community. This membership is required for the protection of other human rights. These reports demonstrate that austerity and its impact has the potential to marginalise individuals, families and communities from the wider political community and society. This marginalization allows for the introduction of further damaging social welfare policies. The impact of these policies is “hiding in plain sight”.

Social workers face two ways at the same time in their fight for social justice (Emirbayer & Williams, 2005; Garrett, 2007). On the one hand, they seek to build positive relationships with individuals, groups, and communities to tackle the barriers to full citizenship. This involves challenging other state bureaucracies or government policy. The greater the distance between the aims of government policies and the stated values of social work, the greater these tensions become. At the same time, social workers are often employed by, or work in, agencies funded by the governments whose policies they oppose or view as unethical. It is, thus, increasingly difficult to categorise the role of a number of state agents in a binary fashion—welfare v. punitive or disciplinary interventions. This has been the case in the period of austerity were welfare agencies have increasingly been forced to ration services. Social workers are classic “street level bureaucrats” (Lipsky, 1980). The complexities of these welfare and other policies are played out in the interactions between individual citizens and the state employees in offices and houses across the country on a daily basis.

Social work and other welfare professions are committed to the key concepts of social justice and human rights. The conception of human rights that is at the heart of their professional values. This conception is a much broader one than that which is the basis of liberal democracy. It is closer to the conception that Somers (2008) outlines. However, social workers and other welfare professionals also function as part of the disciplinary state. Bourdieu et al. (1999, p 184) see these processes as a form of collective “double consciousness” that expose or are “shot through with the contradictions of the State”. This position generates a series of conflicts—social workers and other street level bureaucrats often find themselves in conflict with government policies (Bourdieu, 2005). It would be naive to fail to recognise that welfare regimes are often experienced as bureaucratic and dehumanising (Strier & Binyamin, 2014). For example, Donzelot (1979) noted that poorer families have consistently been subject to greater state surveillance than wealthier ones. The increase in state surveillance will almost inevitably lead to greater intervention. Social work takes place within a specific political and cultural context. As these two reports show, the current one is an environment where poverty and inequality are increasing. Alongside these developments, there is an attack on the fundamental rights of citizenship for marginalised groups. These processes are fundamentally entwined.

## Conclusion

Somers’s (2008) model emphasises the interconnectedness of the market, state and civil society. The institutions of the state and civil society, in this model, should have a key role to play in mitigating the excesses of the market. In this model of a balance of powers between the three elements, civil society can act as a bulwark to protect individuals against excesses of both the state and the market. By doing so, they can create a framework, which helps to ensure that citizens can exercise social, economic, cultural and political rights. Krumer-Nevo (2015) argues that an analysis of poverty and the social work response to it has to start from the proposition that poverty is a violation of human rights. The fissures in UK and Spanish society that the reports outline have been further widened by the impact of the COVID-19 pandemic. The economic impact of the pandemic has pushed more people into poverty and precarious work. In addition, the most marginalised groups are most at risk. These two reports highlight that austerity politics has led to the erosion of the social state and the damage that has caused to individuals, families and communities. The banking crisis of 2008 led to the introduction of austerity policies in the UK and Spain. These policies, particularly in the area of welfare conditionality were an extension of existing trends towards a more punitive welfare approach. However, austerity saw these trends hardened. The most vulnerable were subjected to systems that were based on shame and humiliation. At its core, the welfare retrenchment that occurred under austerity was a denial of the rights of citizenship. The cost of financial instability was paid mostly heavily by vulnerable individuals and communities far removed from the world of speculation in derivatives and other complex financial instruments. Alston (2019a, p22) concluded that “… poverty is a political choice”. The implications of that choice are not just economic, they involve the restriction and denial of fundamental rights to vulnerable citizens.
